# Next Generation Syndromic Surveillance: Molecular Epidemiology, Electronic Health Records and the Pandemic Influenza A (H1N1) Virus

**DOI:** 10.1371/currents.RRN1012

**Published:** 2009-08-22

**Authors:** Raul Rabadan, Neil Calman MD, George Hripcsak

**Affiliations:** ^*^Columbia University; ^†^Institute for Family Health and ^‡^Professor and Chair, Dept of Biomedical Informatics, Columbia University

## Abstract

In the early phase of the 2009 A (H1N1) pandemic a marked increase in severity and a shift in the age distribution toward younger persons was found, with higher severity reported in patients with pre-existing medical conditions and pregnant women. Consistent with previous pandemics, the age and clinical history of the patients play a critical role in the morbidity and mortality associated with the pandemic virus. This is the first influenza pandemic in the information era, where enormous amounts of information will be available from the pathogen and the patient. Recent advances in molecular techniques have provided an enormous amount of information about pathogens in near real time and at relatively low cost. Electronic Health Records (EHRs) provide another enormously rich set of information about patients, which include patient preconditions, previous exposures, immunization history, presenting complaints, duration and severity of illness, treatment history, and geographic location. An infectious disease is a complex interplay between host and pathogen. The morbidity and mortality of a virus depend on the virus, the patient, and the environment. To evaluate and understand the severity of the pandemic virus and to identify the populations at risk of mild or severe, life-threatening illness, it is compulsory to integrate viral and patient information in a fast and accurate way. Both advances in biomedical informatics with the creation of EHRs and molecular techniques provide the framework to achieve these aims.

Since the initial reports in March and April to July 27^th^, 2009, more than 140 countries have reported cases of influenza A (H1N1) to the World Health Organization (WHO) with more than 800 confirmed deaths [1]. In the early phase of the 2009 A (H1N1) pandemic a marked increase in severity and a shift in the age distribution toward younger persons was found [2], with higher severity reported in patients with pre-existing medical conditions and pregnant women [3] [4] [5]. Consistent with previous pandemics, the age and clinical history of the patients play a critical role in the morbidity and mortality associated with the pandemic virus. The reasons for the variable morbidity and mortality are unclear, and several hypothesis need to be considered, including the genetics of the virus, the genetics of the host, clinical history of the patient, secondary infections, and environmental factors.

This is the first influenza pandemic in the information era, where enormous amounts of information will be available from the pathogen and the patient. Recent advances in molecular techniques, including PCR and sequencing, have provided an enormous amount of information about pathogens in near real time and at relatively low cost. Thousands of viral genomes of the pandemic strain have been isolated and sequenced all around the world and have been deposited in databases where scientists can study the origins, the evolution and the increasing diversity of this virus [6] [7] [8] [9]. However, public databases provide little information about the patient.

Electronic Health Records (EHRs) provide another enormously rich set of information about patients, which include patient preconditions, previous exposures, immunization history, presenting complaints, duration and severity of illness, treatment history, and geographic location. At the moment, the severity of an epidemic is usually analyzed retrospectively, through routinely collected surveillance data [2] and by reviewing medical records [3]. These analyses are typically slow, non-automated processes, which have not benefited from the recent advances in health information technologies.

An infectious disease is a complex interplay between host and pathogen and influenced by the vector and ease of transmission. The morbidity and mortality of a virus depend on the virus, the patient, and the environment. To evaluate and understand the severity of the pandemic virus and to identify the populations at risk of mild or severe, life-threatening illness, it is compulsory to integrate viral and patient information in a fast and accurate way. Both advances in biomedical informatics with the creation of EHRs and molecular techniques provide the framework to achieve these aims. 

The implementation of an efficient method for identifying populations at higher risk — especially those at risk of severe sequelae of infection — is a first step to design rapid and effective measures of intervention in the case of a pandemic and of justifying priorities in drug and vaccine delivery and medical attention.

**Figure:**
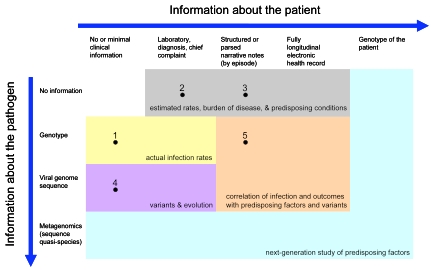
**Figure**. *Spectrum of patient and pathogen information for syndromic surveillance. Shaded regions indicate general goals achievable with the data. Examples of current initiatives are numbered: (1) isolate reporting from reference labs; (2) traditional electronic syndromic surveillance systems; (3) expanded surveillance using structured and narrative EHR data; (4) current sequencing initiatives; and (5) electronic health record-directed genotype sampling.*

******

The Figure represents the spectrum of patient-specific information that can potentially be employed in tracking and understanding H1N1 infection. The intention is that such data would be collected for a population, perhaps under a sampling plan, to elucidate infection rates, to understand organism evolution and spread, and to correlate severity of disease with possible causative or associative factors. Extraction of relevant information from electronic health records is already feasible using structured data elements and, increasingly, with free text data [10] and should become more efficient with increased deployment, improved standards, and use of health information exchange. Recent ARRA legislation [11] provides a mechanism for encouraging the use of health records for surveillance and, in fact, such use is required in the proposed definition of Meaningful Use recommended to the national coordinator that would qualify providers for incentive payments for adoption of EHRs[12].

Pathogen genotyping and sequencing require physical patient contact. Even here, EHRS may be helpful. For example, the Institute for Family Health and the New York City Department of Health and Mental Hygiene have used the automated decision support features of electronic health records at the Institute to encourage obtaining patient viral specimens, instructing providers in the appropriate collection mechanisms and thus allowing the correlation of the pathogen’s genotype or sequence with detailed clinical information.

In the Figure, without specific pathogen information (top row), rates and correlations are presumptive. With the genotype (second row), accurate rates and, with a large enough sample, correlation of predisposing factors may be possible. The large-scale sequencing of viral specimens (third row) would support the study of variants and evolution of disease, and more sophisticated correlations.

As DNA banks grow, the possibility of correlating even patient genotypes (last column) with severity of illness becomes possible. Furthermore, the viral specimen may undergo deep sequencing (fourth row) to determine the microbial diversity, which may be a factor in severity of illness. While this may not be necessary universally, in an appropriately sized sample, such information may bring a greater understanding of a person’s susceptibility to infection.

Not shown in the Figure are anonymous population-level measures like over-the-counter medication purchase rates [13] [14] and Internet searches [15] [16]. Environmental factors are not shown explicitly in the Figure, but are sometimes included in the electronic health record. Alternatively, they could be included as a third dimension in the Figure.

**Acknowledgments**

This work was supported by the Centers for Disease Control and Prevention Center of Excellence grant P01 HK000029 and the Eureka (Exceptional, Unconventional Research Enabling Knowledge Acceleration) grant 1R01LM010140-01.

**Competing interests**

The authors declare that no competing interests exist.
